# Analysis of risk factors for the recurrence of gestational diabetes in subsequent pregnancy: A nationwide population-based study in South Korea

**DOI:** 10.1097/MD.0000000000044044

**Published:** 2025-08-22

**Authors:** Mi Ju Kim, Geum Joon Cho, Jin-Gon Bae, Gi Su Lee, Jeong Ha Wie, Suyeon Park, Won Joon Seong, Hyun Sun Ko

**Affiliations:** a Department of Obstetrics and Gynecology, Kyungpook National University Hospital, School of Medicine, Kyungpook National University, Daegu, Republic of Korea; b Department of Obstetrics and Gynecology, College of Medicine, Korea University, Seoul, Republic of Korea; c Department of Obstetrics and Gynecology, Keimyung University School of Medicine, Daegu, Republic of Korea; d Department of Obstetrics and Gynecology, Eunpyeong St. Mary’s Hospital, College of Medicine, The Catholic University of Korea, Seoul, Republic of Korea; e Department of Obstetrics and Gynecology, Inha University Medical School, Inha University Hospital, Incheon, South Korea; f Department of Obstetrics and Gynecology, Seoul St. Mary’s Hospital, College of Medicine, The Catholic University of Korea, Seoul, Republic of Korea.

**Keywords:** gestational diabetes, recurrence, risk factor

## Abstract

We compared the obstetric and neonatal outcomes of women with and without gestational diabetes recurrence during subsequent pregnancy and identified the risk factors for gestational diabetes recurrence. In this nationwide population-based study, we analyzed pregnant women and their neonates delivered in Korea between January 2015 and December 2021 using the Korea National Health Insurance claims and National Health-Screening Program for Infants and Children databases. In total, 1985,678 pregnant women were analyzed, of whom 15,086 were diagnosed with gestational diabetes in their first pregnancy. By categorizing pregnant women into the gestational diabetes recurrence and nonrecurrence groups, we attempted to confirm the risk factors for gestational diabetes recurrence. Herein, 5672 and 9414 cases of gestational diabetes recurrence and nonrecurrence were analyzed. In the first and second pregnancy, maternal age and gestational age at delivery, and neonatal birthweight, were significantly different between 2 groups (*P* < .001). Preterm birth under 37 weeks of gestation, gestational hypertension, and neonates of large for gestational age, were also significantly more occurred in the recurrence group, compared to the nonrecurrence group (*P* < .001). The interval between the first and second deliveries was significantly longer in the recurrence group (*P* = .0016). In the multivariate analysis, recurrence of gestational diabetes was significantly associated with interpregnancy interval (adjusted odds ratios [OR], 1.008; 95% confidence interval [CI], 1.001–1.016, *P* = .031), presence of gestational hypertension (adjusted OR, 1.206; 95% CI, 1.015–1.432, *P* = .0331), earlier gestational ages at delivery (adjusted OR, 0.913; 95% CI, 0.885–0.942, *P* < .001) and heavier neonates (adjusted OR, 1.134; 95% CI, 1.014–1.268, *P* = .0272), at the first pregnancy. The factors predicting the recurrence of gestational diabetes are interpregnancy interval, gestational hypertension, earlier delivery, and neonates with higher birth weight in the first pregnancy.

## 1. Introduction

Gestational diabetes is a common pregnancy complication, characterized by glucose intolerance and insulin resistance that are initially detected during pregnancy.^[1–3]^ Gestational diabetes is associated with obstetric complications such as gestational hypertension, postpartum bleeding, preterm birth, operative delivery, and maternal mortality.^[1]^ The associated neonatal morbidities include macrosomia, low birth weight, neonatal hypoglycemia, shoulder dystocia or birth trauma, admission to the neonatal intensive care unit, and hyperbilirubinemia.^[1,3–7]^

In gestational diabetes, glucose metabolism is restored to its prepregnancy state at postpartum period, but the risk of type 2 diabetes increases in the long term.^[8,9]^ Additionally, approximately 70% of previously proven gestational diabetes are diagnosed with diabetes 22–28 years after delivery.^[2]^ The incidence of type 2 diabetes is approximately 7.43 times higher in women who had gestational diabetes than in those who did not.^[8]^ Furthermore, these women are prone to cardiovascular complications.^[8]^

The prevalence of gestational diabetes is reportedly 1% to 14%,^[1,2,10,11]^ and the number of cases has recently increased.^[10,12]^ Advanced maternal age, obesity (body mass index [BMI] ≥ 30), family history of diabetes, history of macrosomia delivery, and race/ethnicity (African American, Native American, Asian, and Hispanic) are considerable risk factors for gestational diabetes.^[1]^ A history of gestational diabetes also increases its morbidity in subsequent pregnancies.^[1,13]^ The identification of risk factors for diabetes recurrence in women who had gestational diabetes in previous pregnancies, predicting the likelihood of diabetes recurrence, and provision of preventive education from the beginning of pregnancy may improve the prognosis of diabetes in pregnant women and their neonates.

Therefore, we aimed to compare the basic characteristics and obstetric and neonatal outcomes of women with and without gestational diabetes recurrence in their second pregnancy who had gestational diabetes in their first pregnancy. This study also determined the potential risk factors for gestational diabetes recurrence.

## 2. Methods

### 2.1. Data sources

This nationwide population-based study analyzed pregnant women and their neonates delivered in Korea between January 2015 and December 2021 using the Korea National Health Insurance (KNHI) claims database and National Health-Screening Program for Infants and Children. The majority of Koreans receive health insurance benefits from the Health Insurance Review and Assessment Service. Therefore, the KNHI claims database contains information on the diagnosis and treatment of diseases relevant to most Koreans.

As part of the KNHI healthcare system, a National Health-Screening Program for Infants and Children (NHSP-IC) aged 4 to 80 months undergo a health exam consisting of information on sex, preterm birth, and birth weight. Herein, data were established by merging the KNHI claims and NHSP-IC databases.

### 2.2. Study population

**Figure 1.**
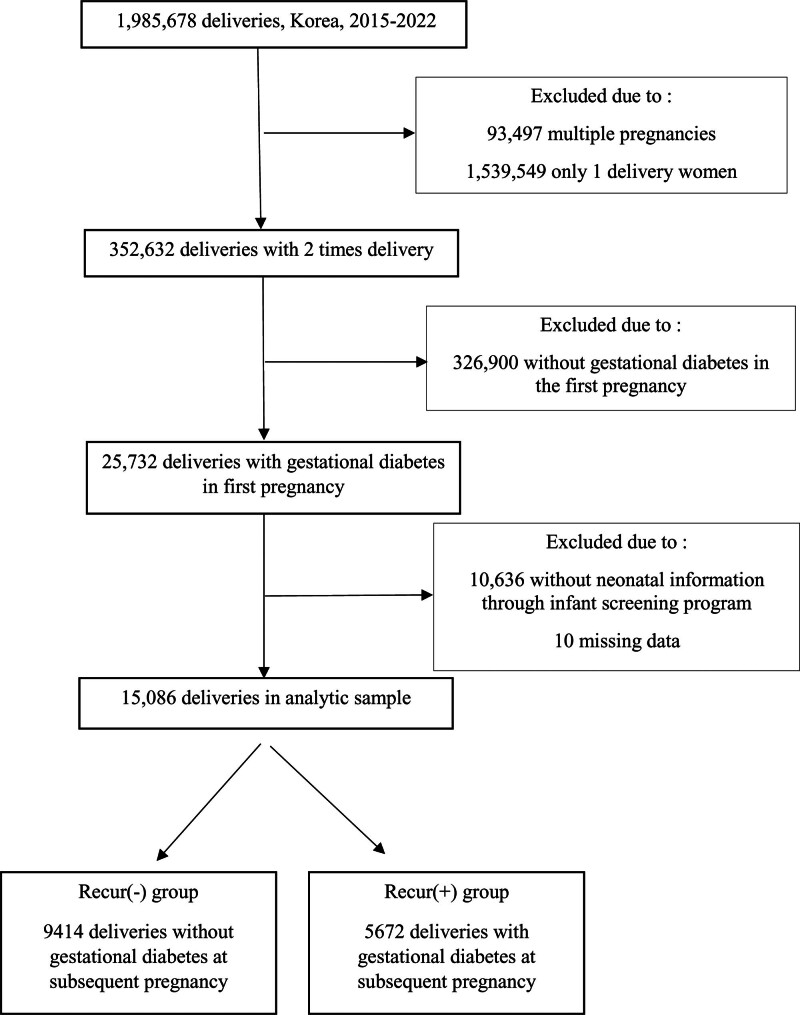
Flowchart of enrollment for study participation.

Among the 1985,678 pregnant women during the aforementioned period, 352,632 who completed 2 pregnancies and deliveries were analyzed, whereas those with multifetal gestation were excluded. Of the remaining sample, 25,732 had gestational diabetes during their first pregnancy. Overall, 15,086 women with available data up to their second pregnancy and whose neonatal information was available through an infant screening program were included in this study (Fig. 1).

### 2.3. Outcomes and covariates

Using the KNHI claims database, gestational diabetes mellitus was diagnosed according to the International Classification of Disease, 10th revision. In Korea, gestational diabetes is diagnosed in accordance with the diagnostic criteria of each center, typically by performing a 50- or 75-g oral glucose tolerance test (OGTT) between 24 and 28 weeks of gestation.^[14,15]^

Covariates such as maternal age and gestational hypertension were identified from the KNHI claims database. Weeks of gestation at delivery, neonatal sex, and birth weight in the first and second pregnancies were determined using the NHSP-IC database. Preterm birth was defined as gestational age <37 weeks. Considering sex and weeks of gestation at delivery, birth weight was calculated as percentile, and weights below the 10th or above 90th percentiles were defined as small (SGA) or large for gestational age (LGA), respectively. The interval between the first and second deliveries was also calculated.

### 2.4. Statistical analysis

The participants were divided into the recurrence and nonrecurrence groups. Continuous variables were expressed as mean ± standard deviation using Student *t* test, whereas categorical variables were expressed as percentages using the chi-squared test. *P* *≤* .05 was considered to indicate statistical significance. Multivariate regression analysis was conducted to determine the risk factors for gestational diabetes recurrence, and the unadjusted odds ratios (ORs) with 95% confidence intervals (CIs) were calculated. All statistical analyses were conducted using SAS version 9.4 (SAS Institute Inc., Cary, NC).

## 3. Results

Among the 1,985,678 pregnant women who delivered between January 2015 and December 2021, 5672 and 9414 with and without gestational diabetes recurrence were compared and analyzed. Of the 15,086 pregnant women diagnosed with gestational diabetes in their first pregnancy, 5672 (37.6%) experienced gestational diabetes recurrence in the subsequent pregnancy.

The maternal characteristics and obstetric and neonatal outcomes of both groups in the first and second pregnancies are presented in Table 1. In the first pregnancy, the recurrence group was older at delivery (32.32 ± 3.59 vs 31.74 ± 3.56 years, *P* < .0001), had a higher frequency of gestational hypertension (4.85% vs 3.4%, *P* < .0001), and tended to have earlier weeks of gestation at delivery (39.38 ± 1.59 vs 39.62 ± 1.47 weeks, *P* < .0001) than the nonrecurrence group. Furthermore, the recurrence group had a higher probability of preterm birth below 37 weeks of gestation (4.04% vs 2.73%, *P* < .0001) and neonatal birth weight (3260 ± 540 vs 3230 ± 490 g, *P* < .0001). The weeks of gestation at delivery were also earlier in the recurrence group, but the birth weight was higher. Therefore, the neonatal weight percentile was possibly higher in the recurrence than in the nonrecurrence group. In addition, the recurrence group had a lower SGA rate (13.52% vs 15.3%, *P* = .0028) but a higher LGA rate (12.29% vs 8.24%, *P* < .0001) and longer intervals between the first and second deliveries (27.95 ± 10.26 vs 27.41 ± 9.99 months, *P* = .0016). In the second pregnancy, the recurrence group was older at delivery (34.65 ± 3.64 vs 34.04 ± 3.65 years, *P* < .0001) and had a higher frequency of gestational hypertension (5.52% vs 3.44%, *P* < .001) than the nonrecurrence group. In the nonrecurrence group, the frequency of gestational hypertension was similar in the first and second pregnancies, whereas in the recurrence group, the frequency was higher in the second pregnancy (5.52% vs 4.85%). The weeks of gestation at delivery tended to be earlier (38.82 ± 1.48 vs 39.07 ± 1.4, *P* < .001), and the rate of LGA was higher (17.97% vs 12.26%, *P* < .001). Even if it was not diagnosed as gestational diabetes in the second pregnancy, the rate of LGA was high at 12.26%, higher than that in women diagnosed with gestational diabetes in their first pregnancy (8.24%).

**Table 1:** Maternal characteristics and obstetric and neonatal outcomes of the first and second pregnancies of women with recurrent and nonrecurrent gestational diabetes.

	Recurrent (‐)	Recurrent (+)	*P*-value
Number	9414	5672	
Interpregnancy interval (mo)	27.41 ± 9.99	27.95 ± 10.26	**.0016** *
First pregnancy			
Age at delivery (yr)	31.74 ± 3.56	32.32 ± 3.59	**<.0001** *
Gestational hypertension, n (%)	320 (3.4%)	275 (4.85%)	**<.0001** *
Gestational age at delivery (wk)	39.62 ± 1.47	39.38 ± 1.59	**<.0001** *
Preterm birth, n (%)	257 (2.73%)	229 (4.04%)	**<.0001** *
Neonatal birth weight (kg)	3.23 ± 0.49	3.26 ± 0.54	**<.001** *
Small for gestational age, n (%)	1440 (15.3%)	767 (13.52%)	**.003** *
Large for gestational age, n (%)	776 (8.24%)	697 (12.29%)	**<.0001** *
Baby male, n (%)	4811 (51.1%)	2824 (49.79%)	.1173
Subsequent pregnancy			
Age at delivery (yr)	34.04 ± 3.65	34.65 ± 3.64	**<.0001** *
Gestational hypertension, n (%)	324 (3.44%)	313 (5.52%)	**<.0001** *
Gestational age at delivery (wk)	39.07 ± 1.4	38.82 ± 1.48	**<.0001** *
Preterm birth, n (%)	315 (3.35%)	283 (4.99%)	**<.0001** *
Neonatal birth weight (kg)	3.27 ± 0.46	3.31 ± 0.52	**<.0001** *
Small for gestational age, n (%)	789 (8.38%)	395 (6.96%)	**.0017** *
Large for gestational age, n (%)	1154 (12.26%)	1019 (17.97%)	**<.0001** *
Baby male, n (%)	4805 (51.04%)	2931 (51.67%)	.4506

* *P* values < .05 are shown in bold with asterisk.

Among women diagnosed with gestational diabetes in their first pregnancy, the risk of recurrence in the next pregnancy was analyzed via multivariate regression analysis (Table 2). A longer interval between the first and second deliveries (adjusted OR, 1.008; 95% CI, 1.001–1.016, *P* = .031). The recurrence of gestational diabetes was higher in cases with gestational hypertension (adjusted OR, 1.206; 95% CI, 1.015–1.432, *P* = .0331), earlier gestational ages at delivery (adjusted OR, 0.913; 95% CI, 0.885–0.942, *P* < .001) and heavier neonates (adjusted OR, 1.134; 95% CI, 1.014–1.268, *P* = .0272).

**Table 2:** Risk of recurrence of gestational diabetes in subsequent pregnancy analysed via multivariate regression analysis. The factors of the first pregnancy were analysed.

	Unadjusted OR	*P*-value	Adjusted OR	*P*-value
Interpregnancy interval (mo)	1.005 (1.002–1.009)	**.015** *	1.008 (1.001–1.016)	**.031** *
Age at delivery (yr)	1.047 (1.037–1.057)	**<.001** *	1.067 (0.983–1.159)	.120
Gestational hypertension, n (%)	1.448 (1.228–1.707)	**<.001** *	1.206 (1.015–1.432)	**.033** *
Gestational age at delivery (wk)	0.903 (0.884–0.923)	**<.001** *	0.913 (0.885–0.942)	**<.001** *
Preterm birth, n (%)	1.499 (1.251–1.797)	**<.001** *	0.924 (0.731–1.167)	.506
Neonatal birth weight (kg)	1.137 (1.066–1.213)	**<.001** *	1.134 (1.014–1.268)	**.027** *
Small for gestational age, n (%)	0.866 (0.788–0.952)	**.003** *	1.051 (0.933–1.328)	.413
Large for gestational age, n (%)	1.56 (1.4–1.738)	**<.001** *	1.147 (0.990–1.328)	.068

* *P* values < .05 are shown in bold with asterisk.

## 4. Discussion

The prevalence of gestational diabetes is widely varied, ranging from 1% to 14%.^[2,11]^ Herein, 25,732 of the 352,632 women who had 2 deliveries were diagnosed with gestational diabetes in their first pregnancy. This indicated an incidence of approximately 7.3%, consistent with that reported in other recent studies.

Furthermore, 5672 of 15,086 (37.6%) women diagnosed with gestational diabetes in their first pregnancy had gestational diabetes recurrence during the subsequent pregnancy. Reportedly, the recurrence rate of gestational diabetes widely varied, from 30% to 84%, which could be attributed to differences in the study population and diagnostic criteria for diabetes.^[11,16]^ Race/ethnicity accounts for a relatively large proportion of the incidence of diabetes.^[4]^ A meta-analysis revealed that non-Hispanic whites had a recurrence rate of 38%, whereas Hispanics, African Americans, and Asians had 56%.^[16]^ Herein, the recurrence rate of gestational diabetes was 37.6%, which was lower than that of other Asian races; this could be attributed to the advanced medical insurance and medical services in Korea and the relatively early management of pregnant women diagnosed with gestational diabetes in previous pregnancies. In a 2008 study in Korea, the recurrence rate of gestational diabetes was 45.0%^[17]^; this reduction could be attributed to the increased availability of information because of the recent provision of education and the efforts dedicated to the prevention of gestational diabetes.^[18]^

The risk factors of gestational diabetes recurrence are advanced maternal age, obesity, insulin use, a family history of diabetes, weight gain of >6.8 kg between their first and second pregnancies, an interpregnancy interval of <24 months, and a high postprandial blood sugar level after OGTT in the first pregnancy.^[16,19–23]^ Parity is also a risk factor for diabetes recurrence, with multiparity reportedly increasing it 3.5- to 4-fold compared with primiparity^[11]^; this is because insulin resistance reduces pancreatic cell function and β-cell reserve decreases with repeated pregnancies.^[24,25]^

Our study demonstrated that the higher the frequency of gestational hypertension during the first pregnancy, the greater the risk of gestational diabetes in the subsequent pregnancy. The recurrence rate in the subsequent pregnancy was approximately 1.2 times higher in women with gestational hypertension than in those without. In gestational diabetes, glucose intolerance and insulin resistance increase blood pressure, which is potentially involved in the pathogenesis of preeclampsia, which increases the risk of preeclampsia by twice or thrice.^[26–28]^ In women with a history of preeclampsia, the probability of gestational diabetes recurrence may increase because of intensified insulin resistance and dysfunction of pancreatic β-cells in subsequent pregnancy.^[26–28]^

In this study, the longer the interval between pregnancies, the higher the risk of diabetes recurrence; it is consistent with the interpregnancy interval of ≥36 months in previous studies.^[4]^ However, this is contrary to the finding of a high risk of recurrence within 24 months between pregnancies.^[19]^ The authors explained that shorter intervals are less likely to promote weight loss after the first delivery; thus, the recurrence is high because BMI is higher before the second pregnancy. Furthermore, in this study, the interpregnancy interval for both groups was over 27 months. Long interpregnancy intervals increase maternal age at delivery, which may induce gestational diabetes recurrence.

The recurrence of diabetes was analyzed to be related to baby’s birthweight at the first delivery. In univariate analysis, it was confirmed that there was a relationship between SGA or LGA with gestational diabetes recurrence, but multivariate analysis was analyzed to be less. Other studies have not identified the relationship between SGA or LGA in their first pregnancy and gestational diabetes in the subsequent pregnancy, too.^[20]^ Compared with the general population, the frequency of macrosomia was 12% higher in women who were previously diagnosed with gestational diabetes but did not experience a recurrence in the subsequent pregnancy; this can be considered when women are older or are not diagnosed with gestational diabetes during OGTT; however, it leads to gestational diabetes as pregnancy progresses.

This study has some limitations. First, it was based on data from the KNHI claims and NHSP-IC databases, and there is no detailed information on insulin use, maternal BMI, or demographic characteristics of pregnant women and their neonates. Second, inconsistent diagnostic methods and criteria for gestational diabetes were used. Most hospitals used a 2-step diagnosis based on 50- and 100-g OGTT, but in some cases, 1 step of 75-g OGTT might have been used. Nevertheless, the strengths of this study are that it included a large number of study participants, provided an overview of gestational diabetes and recurrence over a specific period, and demonstrated that gestational hypertension and earlier delivery—rarely reported in other studies—are associated with gestational diabetes recurrence. Furthermore, the effects of gestational diabetes can be better understood by comparing groups with and without gestational diabetes recurrence and analyzing the long-term prognosis for mothers along with the development of metabolic diseases in neonates over time.

## 5. Conclusions

Factors predicting the recurrence of gestational diabetes are gestational hypertension, earlier delivery, and neonates with higher birth weight in the first pregnancy. Having advanced knowledge of these risk factors and providing appropriate counseling to women with gestational diabetes who are planning their next pregnancy are considered helpful in preventing gestational diabetes recurrence and in the treatment and prognosis of mothers and neonates with gestational diabetes.

## Author contributions

**Data curation:** Geum Joon Cho, Mi Ju Kim, Gi Su Lee, Jeong Ha Wie, Suyeon Park.

**Methodology:** Jin-Gon Bae, Geum Joon Cho.

**Writing – original draft:** Mi Ju Kim, Geum Joon Cho.

**Writing – review & editing:** Won Joon Seong, Hyun Sun Ko.
